# Extraction of Gelatin From Poultry Byproduct: Influence of Drying Method on Structural, Thermal, Functional, and Rheological Characteristics of the Dried Gelatin Powder

**DOI:** 10.3389/fnut.2022.895197

**Published:** 2022-06-10

**Authors:** Jahangir A. Rather, Syed Darakshan Majid, Aamir Hussain Dar, Tawheed Amin, H. A. Makroo, Shabir Ahmad Mir, Francisco J. Barba, B. N. Dar

**Affiliations:** ^1^Department of Food Technology, Islamic University of Science and Technology, Pulwama, India; ^2^Department of Food Science and Technology, SKUAST-K, Srinagar, India; ^3^Department of Food Science and Technology, Government College for Women, Srinagar, India; ^4^Nutrition and Food Science Area, Preventive Medicine and Public Health, Food Science, Toxicology and Forensic Medicine Department, Faculty of Pharmacy, Universitat de València, Burjassot, Spain

**Keywords:** gelatin, byproduct, poultry, drying, extraction, flow behavior

## Abstract

The poultry processing industrial wastes are rich sources of gelatin protein, which can be utilized for various industrial sectors. The present investigation was conducted to evaluate the effect of freeze-drying (FD) and hot air drying (HAD) on the physicochemical, structural, thermal, and functional characteristics of chicken feet gelatin. The yield (%) of extracted FD and HAD gelatin was 14.7 and 14.5%, respectively. The gelatin samples showed lower percent transmittance in the UV region. The FTIR bands were at 3,410–3,448 cm^−1^, 1,635 cm^−1^, 1,527–334 cm^−1^, and 1,242–871 cm^−1^ representing amide-A, amide-I, amide-II, and amide-III bands, respectively. The water activity of HAD was higher (0.43) than in FD (0.21) samples and pH were 5.23 and 5.14 for HAD and FD samples, respectively. The flow index (*n*) of 6.67% gelatin solutions was 0.104 and 0.418 with consistency coefficient (*k*) of 37.94 and 31.68 for HAD and FD samples, respectively. The HAD sample shows higher gel strength (276 g) than the FD samples (251 g). The foaming capacity (FC) and foaming stability (FS) of FD samples were 81 and 79.44% compared to 62 and 71.28% for HAD, respectively. The emulsion capacity and emulsion stability of HAD gelatin were higher at 53.47 and 52.66% than FD gelatin. The water holding capacity (WHC) and oil binding capacity (OBC) of FD were lower, that is, 14.3 and 5.34 mL/g compared to HAD gelatin having 14.54 and 6.2 mL/g WHC and OBC, respectively. Hence, the present study indicated that gelatin samples can be utilized in various food products for enhancing functionality and can be used for developing edible packaging materials.

## Introduction

Food industries produce vast amounts of processing wastes which may be starch, protein, or lipid-based. Among various food processing industries, the poultry industry sector has shown spectacular growth in India since the late 70s due to the higher consumption of poultry meat. South and West Indian states have been the leading states in this regard (1). A huge amount of poultry waste generated can cause environmental pollution problems if not utilized properly, although these wastes are rich sources of protein that can be utilized in food industries for various functionalities (2). Gelatin, an essential ingredient of poultry waste and byproducts, obtained by partial collagen hydrolysis, is a high molecular weight, hot water-soluble protein and has gel-forming capacity. Determination of physicochemical, thermal, structural, textural, and functional characteristics of the gelatin is important in knowing the applicability of extracted gelatin (3). Gelatin is famous for its numerous functionalities in the food, packaging, and drug industries, acting as a stabilizer, thickener, texturing agent, and an essential packaging ingredient (3). It is used in the food product manufacturing process as a thickener in sauces and meals to provide creamy consistency. It is furthermore incorporated in low-fat spreads to act as binding agents and makes the product more suitable for health-conscious people. Gelatin hydrolysate has also been incorporated in various energy drinks for athletes due to their need of higher energy for performing sports activities. The gelatin hydrolysates from numerous sources, such as the skin of sole, squid, and cobia, and the poultry waste gelatin has also been assessed for its functional and antioxidant properties (4).

The functional and physicochemical properties of gelatin recovered from food waste and byproducts get influenced by the technique of extraction used and post-extraction factors, such as processing conditions which are drying technique used, chemicals and time-temperature combinations utilized, and the method of drying used for drying the extracted gelatin. The various drying methods used in the production of gelatin consist of lyophilization (near −50°C), vacuum drying, and hot air (>45°C) drying (4). Among these different drying methods, lyophilization is gaining importance due to its high-quality retention property in the products to be dried. The lyophilized gelatin is extensively used for high-quality food and pharmaceutical materials, like proteins, vaccines, bacterial, and mammal cells for its high quality (5). In vacuum drying, the drying temperature is also low which gives the product a higher quality than the conventional hot air drying technique (6). The thermal damage caused by higher drying temperatures alters the functional properties of the product owing to denatured molecule aggregation. In addition, high temperature results in the production of low molecular peptides. The low molecular peptides easily hydrolyze compared to peptides of higher molecular weight (3). This may alter the properties of the recovered gelatin and hence can affect the functional property of gelatin, as gelatin is used in food, pharmaceutical, and packaging industries for numerous functionalities.

Hence, the purpose of the current investigation was to obtain gelatin from poultry wastes (feet) for better poultry waste utilization. The effect of drying techniques (freeze-dried and hot air dried) on the physicochemical, structural, thermal, and functional properties, such as foaming capacity/stability, emulsion capacity/stability, water holding capacity, and oil holding capacity of the extracted gelatin, were also evaluated to study the functionality of, in particular, dried gelatin in specific industrial sector.

## Materials and Reagents

Fresh chicken feet were obtained from Lassipora Pulwama poultry slaughtering unit. The chicken feet were then instantaneously transported to the laboratory, washed, denailed, and then stored in the refrigerator till further processing. The reagents were of analytical grade and include lactic acid (Nice Chemicals Pvt. Ltd., Kerala, India), sodium hydroxide, NaOH (Qualikems, Pvt. Ltd, India), and HCl (Qualikems, Pvt. Ltd., India).

### Gelatin Extraction From Chicken Feet

Gelatin was obtained from poultry feet as per the technique adopted by Chakka et al. (7), with minor modification. The denailed feet were then sliced into small chunks and grounded in a grinder to form a paste. The paste was mixed with 0.5 M NaOH solution and kept as such for 2 h. The blend was then filtered using a muslin cloth and then treated with 10% butyl alcohol to remove fat. The defatted sample was then treated with HCl (0.1 N) solution (1:6 w/v) and stirred continuously for 24 h to remove inorganic compounds followed by filtration and washing with tap water. The sample was then treated with 4.5% lactic acid solution (1:1) placed overnight and heated (55°C) in a water bath (20 min) for gelatin extraction. Post-incubation sample was filtered using a muslin cloth and then with filter paper (Whatman, 4) by Buchner funnel, and the filtrate was collected for drying.

### Drying and Yield of Gelatin

The extracted gelatin was dried using two methods namely, hot air drying (HAD) and freeze-drying (FD). In the hot air method of drying, gelatin was dried at 45°C in a tray drier (Tray drier, SSI-103C). The freeze-drying was performed at −60°C temperature and pressure of 30 Pa using a freeze drier (Bio-base, BK-FD10P). The dried samples were ground to powder and then placed in the glass bottles for further analysis. The yield percentage was calculated using Equation (1).


(1) $$\begin{array}{l} \text{per cent Yield percent}=\frac{\text{Weight of powdered gelatin }\left(\text{g}\right)}{\text{wet sample weight }}\times 100 \end{array}$$

### Gelatin Proximate Composition

The composition of hot air and freeze-dried gelatin was determined by AOAC (7) methods. The moisture content of FD and HAD gelatin was done at 105°C using oven-drying method. The Kjeldahl method was used for the determination of the protein content of samples (Foss Kjeltec™ 8200) using a conversion factor of 5.55 similar to the method of Kanwate and Kudre (8). The ash content of FD and HAD samples was determined at 550°C using a muffle furnace overnight. The total fat was determined by the standard method (7).

### Light Transmittance of Gelatin Solutions

The light transmittance of all gelatin solutions was determined as per the method by Hazirah et al. (9) using a double beam UV-Visible spectrophotometer (UV-VIS, L1-2904) in the 200–800 nm wavelength range.

### FTIR Analysis of Gelatin Solutions

The FTIR spectral analysis of HAD and FD gelatin samples was determined by FTIR spectrometer Spectrum 2 (L1600300, Perkin Elmer). The FTIR spectra were obtained at 400–4,000 cm^−1^ range at 4 cm^−1^ resolution with 32 scans at 25°C (8).

### DSC Analysis of Gelatin Samples

The DSC analysis of the samples was performed using Perkin Elmer, DSC, 8000. In brief, 4 mg gelatin powder was heated with a heating rate of 5°C/min from −20 to 200°C in the presence of nitrogen gas.

### Instrumental Color

The color analysis of both HAD and FD gelatin samples was determined using Hunter Lab Colorimeter (Flex EZ Model No. 45/0). The L^*^, a^*^, and b^*^ values of the samples were determined in triplicates.

### Estimation of pH Values

Gelatin solution was developed by dissolving 1 g gelatin at 60°C in 100 mL distilled water. The solution was then cooled to 23 ± 2°C temperature. The pH was determined by a digital pH meter (LABMAN LMPH-12, India), and before the measurement, the equipment was calibrated with a buffer of pH 4, 7, and 9.1.

### Water Activity of Gelatin Samples

The water activity of both hot air dried and freeze-dried samples were done in triplicates using a water activity meter (Novisina AG CH-8853 Lachen) at 25°C.

### Rheological Properties

Gelatin solutions preparation for both FD and HAD was done by dissolving 6.67 g of gelatin in purified water and making the total volume 100 mL using a hot plate stirrer (45°C). The viscoelastic properties of gelatin solutions were determined using dynamic oscillatory measurements similar to the method of Rasid et al. (10), with slight alterations using parallel plate geometry (50 mm diameter) of rheometer (Physica MCR 101 Anton Paar). The linear viscoelastic range (LVR) was obtained by performing an amplitude sweep (0.1–20%) at 1 rad/s frequency. The 1% strain amplitude within the LVR domain was selected for performing a frequency sweep at 25°C from 0.1 to 100 rad/s for each gelatin sample solution. The viscoelastic parameters determined were the G′ and G″. The flow behavior of these samples was determined at 0.1–100 rad/s at 25°C. The temperature sweep tests were determined from 10 to 40°C with constant heating of 2°C/min.

### Texture Profile Analysis

TPA was determined by TA-TX2 texture analyzer (TA-HD plus, 5213 Surrey, England), using a load cell (50 kg). The test conditions were similar to that of Chandra and Shamasundar's (11) method. Gelatin gels (6.67%) were developed by dissolving the gelatin powder in distilled water followed by continuous stirring (30 min) using a magnetic stirrer at 50–60°C. The gelatin solutions were then kept at (4–6°C) in the refrigerator for 24 h. The gelatin gels were then used for TPA analysis. The parameters to be determined were hardness, springiness, gumminess, adhesiveness, cohesiveness, and chewiness.

### Gel Strength Determination

The gel strength (bloom strength) of gelatin-developed gels was determined using TAXT Texture Analyzer (TA-HD plus, 5213, Surrey, England), with a 5 kg load cell, crosshead speed (1 mm/s), and a flat bottomed plunger with 0.5 inch diameter. The (6.67%) gelatin solution was prepared in bloom jars. The gelatin solutions were heated for 30 min at 60°C and then were set aside at 7–8°C in the refrigerator (16–18 h). The penetration test was performed by placing the bloom jar centrally under the plunger. The maximum force in grams was determined by penetrating the probe up to 4 mm depth.

### Foaming Capacity and Foaming Stability Properties

The FC and FS of FD and HAD gelatin samples similar to the technique of Sathe et al. (12). One gram of gelatin for both FD and HAD was weighed and placed in centrifuge tubes and 50 mL of distilled water was added to each tube and then tubes were heated at 60°C to dissolve it completely. The gelatin solutions were then homogenized at 10,000 × g for 5 min for foam development. These homogenized solutions were then poured into a measuring cylinder for the determination of foaming capacity and stability using Equations (2) and (3) as follows:


(2) $$\begin{array}{l} \text{Percent foaming capacity}=\frac{\text{Foam volume }-\text{Initial liquid volume}}{\text{Initial liquid volume}}\times 100 \end{array}$$

(3) $$\begin{array}{l} \text{Foaming stability }\left(\text{\%}\right)=\frac{\left(\text{Foam volume after }30\text{ min}-\text{Initial liquid volume}\right)}{\text{Initial liquid volume}}\times \;100 \end{array}$$

### Water Holding Capacity

The water holding capacity (WHC) of FD and HAD gelatin samples was determined by the method of Rasli and Sarbon (13) with minor alterations. In brief, 0.5 g of gelatin samples for each sample was dissolved in distilled water (10 mL) in centrifuge tubes. The gelatin solutions were then vortexed for 30 min and centrifuged for 25 min at 2,800 × g. The supernatant was filtered by filter paper (Whatman, no. 1) and the volume of the supernatant was measured. The variance between the initial volume of water used and the supernatant volume was determined. The WHC of the samples was calculated using Equation (4) as follows:


(4) $$\begin{array}{l} \text{WHC }\left(\text{mL}/\text{g}\right)=\frac{\text{Initial volume}-\text{volume of supernatant}}{\text{Weight of gelatin }\left(\text{g}\right)}\times 100 \end{array}$$

### Oil Binding Capacity

The oil binding capacity (OBC) of FD and HAD gelatin samples was determined similar to the method of Shahidi et al. (14). The gelatin sample (0.5 g) was taken in centrifuge tubes for each sample and 10 mL sunflower was added to each tube followed by vortexing for 30 min. The samples were then centrifuged for 25 min at 2,800 × g, then the oil was emptied and OBC was determined using the Equation (5) as follows:


(5) $$\begin{array}{l} \text{Oil binding capacity }\left(\frac{\text{mL}}{\text{g}}\right)=\frac{\text{Initial volume}-\text{volume of supernatant}}{\text{Weight of gelatin taken}}\times 100 \end{array}$$

### Emulsifying Capacity and Stability

The emulsion capacity (EC) and emulsion stability (ES) of FD and HAD gelatin samples were determined using the method of Bichukale et al. (15). The emulsions of samples were prepared using gelatin (1 g), distilled water (50 mL), and sunflower oil (50 mL). These solutions in centrifuge tubes were then dispersed using a homogenizer. For determining emulsion capacity, the centrifuge tube was centrifuged for 10 min at 4,000 × g, while for determining emulsion stability, the sample was homogenized under the same conditions and then heated at 80°C in a water bath for 30 min. The samples were then cooled to 25°C and the emulsion capacity and emulsion stability were determined using Equations (6) and (7) as follows:


(6) $$\begin{array}{l} \text{Emulsion capacity }\left(\text{\%}\right)=\;\frac{\text{Emulsion layer height}}{\text{Whole layer height}}\times 100 \end{array}$$

(7) $$\begin{array}{l} \text{Emulsion stability }\left(\text{\%}\right)=\frac{\text{Emulsion layer height after heating at }80^{{}^{\circ}}\text{C}}{\text{Hight of whole layer}}\times 100 \end{array}$$

### Statistical Analysis

All the tests in the study were performed and the values are reported as the mean of triplicates. Data analysis for analysis of variance (one-way ANOVA) was done by using SPSS statistics software (v.16, SPSS Inc., Chicago, IL, USA). The significance of the data points was identified by *post hoc* Duncan's test at a significance level of 5% (*p* < 0.05).

## Results and Discussions

### Effect of Drying Methods on Gelatin Yield

The HAD and FD yields of gelatin obtained from poultry feet by 4.5% lactic acid were 14.5% (16.95% db) and 14.7% (17.23% db), respectively. The gelatin yield in the FD sample was slightly higher than in the HAD samples. Kanwate et al. (16) also reported a higher yield of gelatin in freeze-drying than spray and vaccum drying methods, and that the lower yield in other drying techniques other than freeze-drying is likely due to sticking of gelatin powder to the walls which could not be recovered from dryers. Our results are in accordance with Widyasari and Rawdkuen (17), the yield of gelatin from chicken feet by acidic methods in their study was 12.64 and 12.37% in ultrasonic-assisted methods. This variation in the gelatin recovery may be owing to the collagen loss by leaching through washing or by inappropriate hydrolysis of collagen (14). The variation in gelatin yield may also get altered by species, chicken age, proximate compositions, and used gelatin extraction methods (15).

### Proximate Composition of Gelatin

The chicken feet gelatin (HAD and FD) proximate composition is displayed in Table 1. There is a non-significant variance between FD and HAD samples in proximate composition (*p* > 0.05) except for the moisture content. The moisture content in HAD gelatin was slightly higher than in FD. In a study by Almeida and Lannes (18), the moisture, protein, fat, and ash content of chicken skin and tendons were 10.39, 84.96, 2.71, and 1.91%, respectively. In addition, Sarbon et al. (19) reported 9.68, 81.75, and 1.06% moisture, protein, and ash contents, respectively, of commercial bovine gelatin. The moisture and fat contents of both HAD and FD gelatin are lesser than in the studies conducted by Almeida and Lannes (18), which confirms that pretreatments and drying methods efficiently reduced fat and moisture, hence enhances keeping quality of extracted gelatin. The protein content of the present study was in close agreement with Widyasari and Rawdkuen (17), who reported 90.06% protein content of gelatin extracted by acidic methods.

**Table 1:** Yield, proximate composition, pH, and water activity of HAD and FD gelatin.

**Chicken feet gelatin**	**Hot air dried**	**Freeze dried**
Yield (%)	14.50 ± 0.02^a^	14.70 ± 0.01^b^
Moisture (%)	5.41 ± 0.01^b^	5.37 ± 0.03^a^
Protein (%)	90.29 ± 0.02^a^	90.27 ± 0.01^a^
Fat (%)	1.50 ± 0.02^a^	1.53 ± 0.04^a^
Ash (%)	2.80 ± 0.01^a^	2.84 ± 0.04^a^
pH	5.23 ± 0.02^b^	5.14 ± 0.01^a^
Water activity	0.43 ± 0.01^b^	0.21 ± 0.01^a^

*The values are presented as mean ± standard deviation (n = 3), the data with different superscripts in same rows are significantly different (p < 0.05)*.

### Physico-Chemical Properties

#### Color, pH, and Water Activity

The pH and water activity values of the HAD and FD gelatin samples are presented in Table 1. The pH values did not differ significantly in HAD and FD gelatin samples. The pH values depend on the process used for the extraction of gelatin (20). Since the extraction procedure for both the gelatin samples is the same, there is no significant difference in their pH values. Water activity values of HAD and FD samples are 0.43 and 0.21, respectively. The pH values of 7.35, 6.55, 8.16, and 5.40 were reported by Singh et al. (21) of tray dried, freeze-dried, drum dried, and commercial dried gelatin samples, respectively. The lower water activity of FD samples shows that it is a better drying method and can prolong the shelf-life of the powder. da Silva Araújo et al. (22) also reported the water activity (0.20) of fish skin gelatin.

The color values of HAD and FD samples were shown in Table 2. The color value of gelatin is dependent on raw material and conditions of extraction. The color attribute does not have any effect on the functional properties of gelatin but has an impact on its consumer acceptability (23). The color is characterized by L^*^, a^*^, and b^*^ values. The drying method was found to affect the color of chicken feet gelatin significantly (*p* < 0.05) as shown in Table 2. Both FD gelatin powder, as well as the FD gelatin solution, had higher lightness (L^*^) values as compared to their HAD counterpart, respectively. Kim et al. (24) also reported higher L^*^ and lower a^*^ and b^*^ values of FD than vacuum-dried yellow croaker fish samples. The lower lightness (L^*^), higher redness (a^*^), and yellowness (b^*^) values of HAD gelatin samples are owing to the Maillard reaction that occurred at higher temperatures in the tray drying process. Maillard's reaction is due to the reaction between released free amino acids and free C=O groups of gelatin (18).

**Table 2:** Texture profile analysis of freeze dried and hot air-dried gelatin.

**Characteristics**		**Gelatin type**	**Hot air dried**	**Freeze dried**
TPA values		Hardness (N)	38.73 ± 0.01^a^	12.57 ± 0.01^b^
		Adhesiveness (N)	0.013 ± 0.01^a^	0.0576 ± 0.01^b^
		Cohesiveness	0.005 ± 0.002^a^	0.003 ± 0.001^b^
		Gumminess (N)	16.86 ± 0.02^a^	3.72 ± 0.04^b^
		Chewiness (N)	5.22 ± 0.03^a^	1.23 ± 0.03^b^
		Springiness (mm)	0.0028 ± 0.001^a^	0.0032 ± 0.001^a^
Color values	Powder	L*	78.49 ± 0.03^b^	89.96 ± 0.01^a^
		a*	1.73 ± 0.01^a^	0.20 ± 0.03^b^
		b*	25.06 ± 0.02^a^	10.09 ± 0.01^b^
	Solution	L*	35.87 ± 0.02^b^	42.34 ± 0.01^a^
		a*	2.74 ± 0.03^a^	2.52 ± 0.02^b^
		b*	0.43 ± 0.02^b^	9.62 ± 0.01^a^

*The data are presented as mean ± standard deviation (n = 3), the data with different superscripts in rows are significantly different (p < 0.05)*.

#### Transmittance (%) of Freeze-Dried and Tray Dried Gelatin Samples

Determination of percent transmittance of gelatin samples is important, as the gelatin is an essential ingredient for the development of edible films and coatings. Lower transmittance of gelatin samples in the UV region indicates the presence of higher aromatic amino acids, and hence better suited for the packaging industry for the development of biodegradable films for enhancing shelf-life of food products (25). Transmittance values of FD and HAD samples are presented in Table 3. It is evident that for both HAD and FD gelatin sample solutions with different concentrations of gelatin (1–4%), the transmittance values get increased with changing wavelength from 200 to 800 nm. The transmittance values for both HAD and FD gelatin solutions were lowest at UV wavelengths (200 and 280 nm). The lower transmittance (higher UV barrier) properties of both gelatin samples are may be due to higher amounts of aromatic amino acids (24). With increasing concentration of gelatin from 1 to 4%, the light transmittance of both gelatin samples decreases. These results suggest that gelatin has a protective effect against lipid oxidation and hence gelatin is suitable for the edible coating and film development for food packaging applications. These results are in line with Ahmed et al. (26), showing gelatin films have UV barrier properties.

**Table 3:** Transmittance values of HAD and FD gelatin solutions (1–4%).

**Samples**	**Concentration**	**Wavelength (nm)**							
		**200**	**280**	**350**	**400**	**500**	**600**	**700**	**800**
		**Transmittance (%)**							
Freeze dried	1%	0.01	0.01	1.68	2.08	2.65	6.77	9.17	11.75
	2%	0.01	0.01	0.98	0.42	0.58	1.65	2.48	3.58
	3%	0.01	0.01	0.42	0.23	0.57	0.93	1.29	1.68
	4%	0.01	0.01	0.21	0.01	0.37	0.58	0.74	0.94
Hot air dried	1%	0.01	0.01	14.02	23.75	38.89	46.83	58.4	65.11
	2%	0.01	0.01	2.68	6.31	14.9	24.02	32.68	40.02
	3%	0.01	0.01	1.04	2.79	8.33	15.59	23.36	30.68
	4%	0.01	0.01	0.01	1.79	5.51	7.37	17.28	23.88

#### Fourier-Transform Infrared

**Figure 1:**
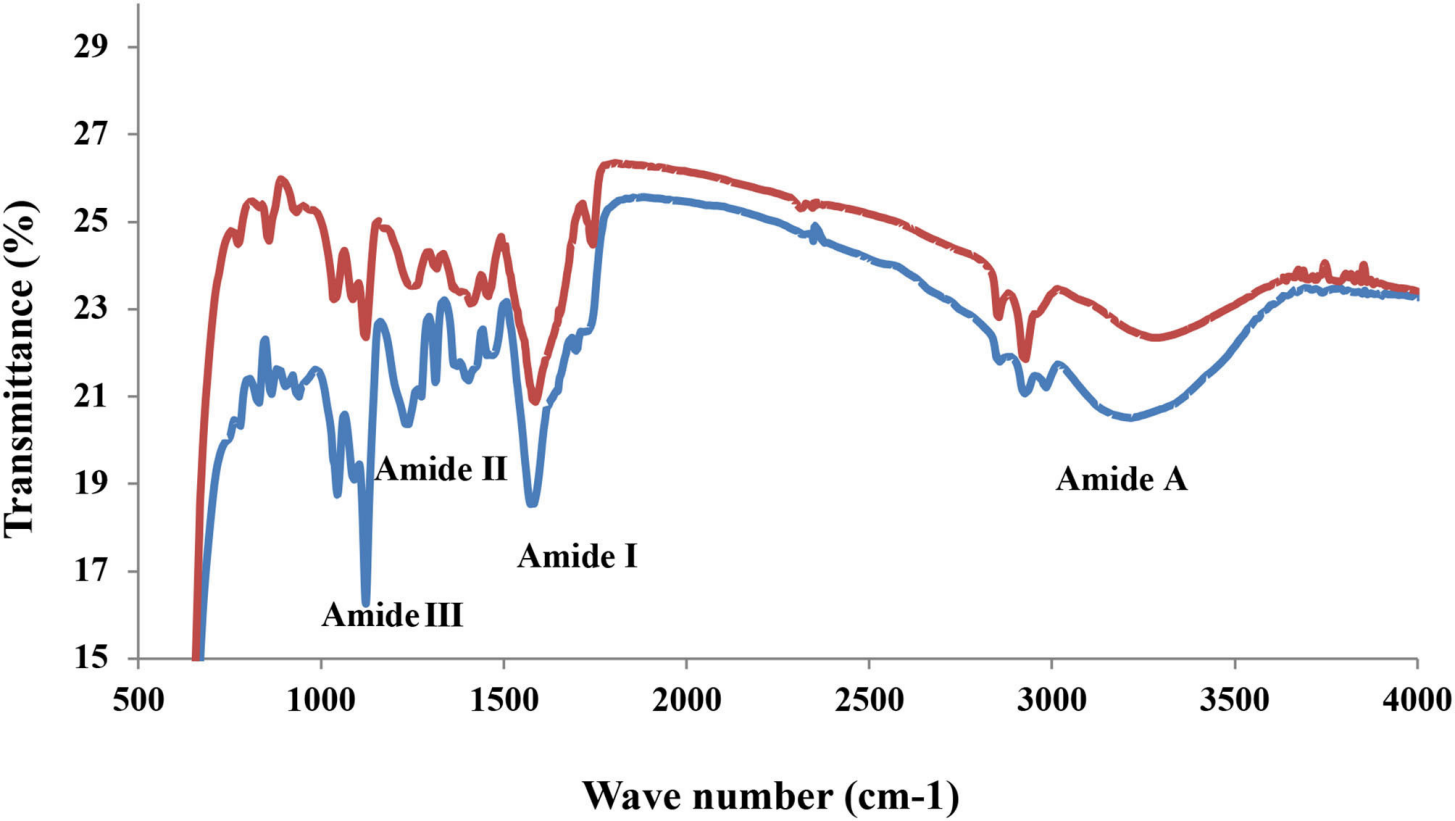
FTIR spectra of FD (**–**) and HAD (**–**) gelatin.

The FD and HAD sample FTIR spectra are shown in Figure 1. The FTIR analysis is used to find the secondary structure and functional groups present in gelatin (21). The FTIR analysis of FD and HAD gelatin samples showed four peaks in the amide regions. The 3,410–3,448 cm^−1^ represents amide-A, 1,635 cm^−1^ represents amide-I, 1,527–1,334 cm^−1^ represents amide-II, and 1,242–871 cm^−1^ represents amide III bands. The amide-A band is owing to stretching NH vibrations, indicating gelatin-coiled structure, since stretching of free NH group vibrations is generally observed at 3,400–3,440 cm^−1^ (22). These vibrations are beneficial for the analysis of protein secondary structure in the IR spectral region (18). The amide II peaks in the gelatin samples are attributed to an out-of-phase CN stretch with in-plane NH deformation combination styles of the peptide groups, whereas amide III of gelatin samples is indicative of disorders in the molecules of gelatin and are possibly allied with the triple-helix structure loss (22). Kanwate et al. (16) reported Amide-A at 3,294–3,273 cm^−1^, Amide-B at 2,919, 2,928, and 2,919 cm^−1^, Amide-I at 1,632.93, 1,634.36, and 1,631.50 cm^−1^, Amide-II at 1,537, 1,529, and 1,524 cm^−1^ and Amide-III bands at 1,236, 1,239, and 1,236 cm^−1^ for freeze-dried, spray dried, and vacuum dried samples fish gelatin samples, respectively.

#### Thermal Analysis (DSC)

**Figure 2:**
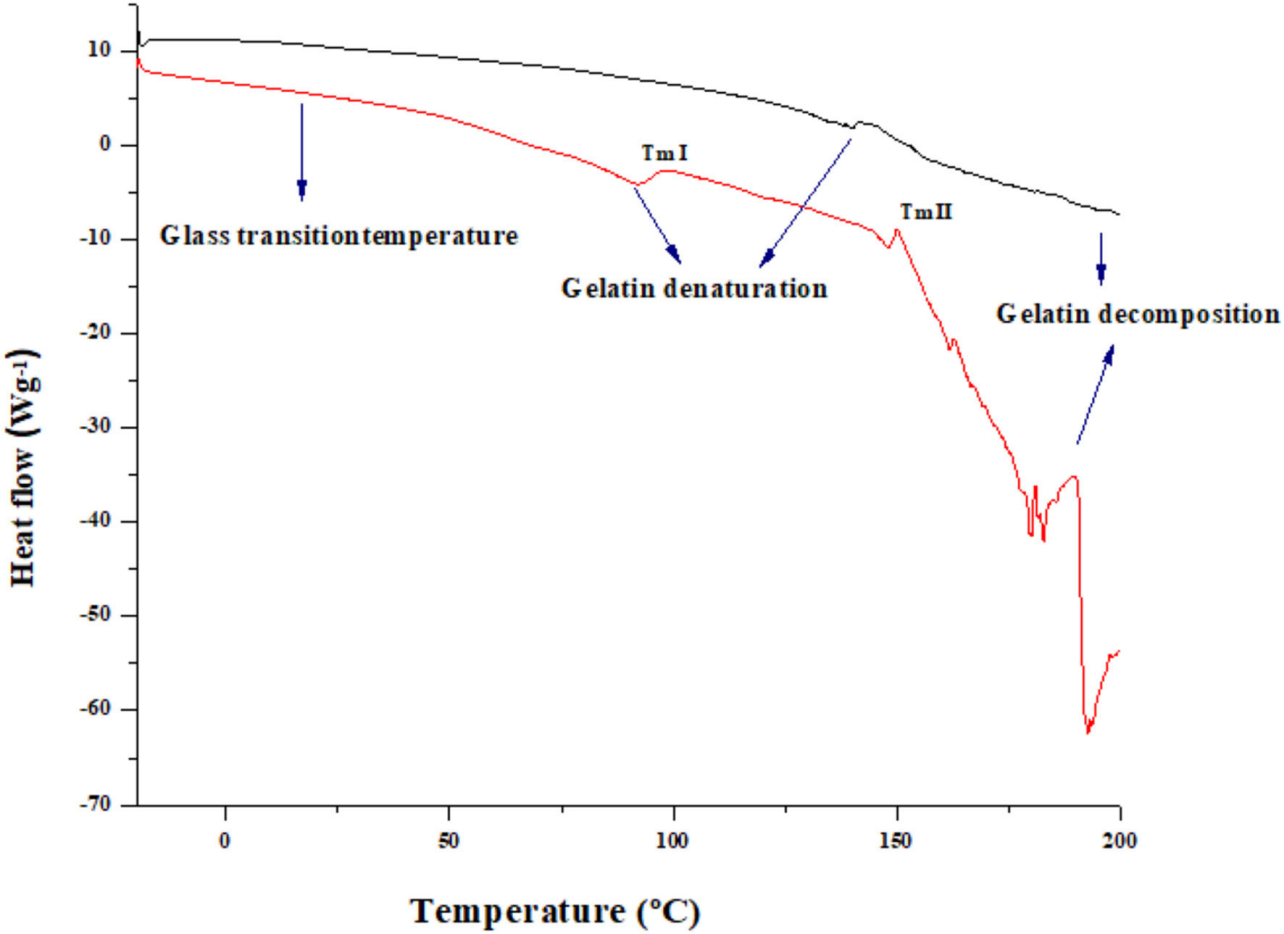
The DSC analysis of HAD (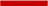) and FD (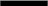) samples.

The DSC analysis acts as a conspicuous tool in investigating the thermal properties of the gelatin (25). The DSC graph of both HAD and FD samples is shown in Figure 2. The broad region in DSC curve gelatin powder, at a temperature of 148.93 for HAD and 139.94 for (FD) samples, with the onset temperature at 92.05 and 91.891°C in case of FD and HAD samples, respectively, is not clearly visible in FD sample. This authorizes the results of several further authors and is related to the loss of water and the glass transition temperature (Tg) owing to the transition of the random coil from the triple helix (25). The glass transition was followed by an endothermic peak which is accredited to the triple-helix crystalline structure melting. This change is linked with the melting and detachment of ordered regions. Numerous authors ascribed this endothermic peak to the coinciding of diverse processes like the evaporation of water, recrystallization and melting of smaller or imperfect crystallites of gelatin, and connotation of glass transitions of polypeptide chain α-amino acid blocks (27). The two melting temperatures shown by HAD samples consist of melting temperature I and melting temperature II. The melting temperature I indicates that molecules obtain the freedom of motion to spontaneously change into crystalline form. However, the melting temperature II indicates the temperature above which the polymer chains can move freely. Al-Saidi et al. (28) reported that the two curve line shifts specify two types of existing amorphous regions in samples at a particular temperature. A transition was also observed around 181.41–200.5°C for FD and 189.95–200.89°C for HAD gelatin samples and represents polymer decompositions owing to the peptide bond breakage (27). These results indicate that HAD gelatin samples have higher thermal stability than FD samples.

### Rheological Properties

#### Flow Behavior

**Figure 3:**
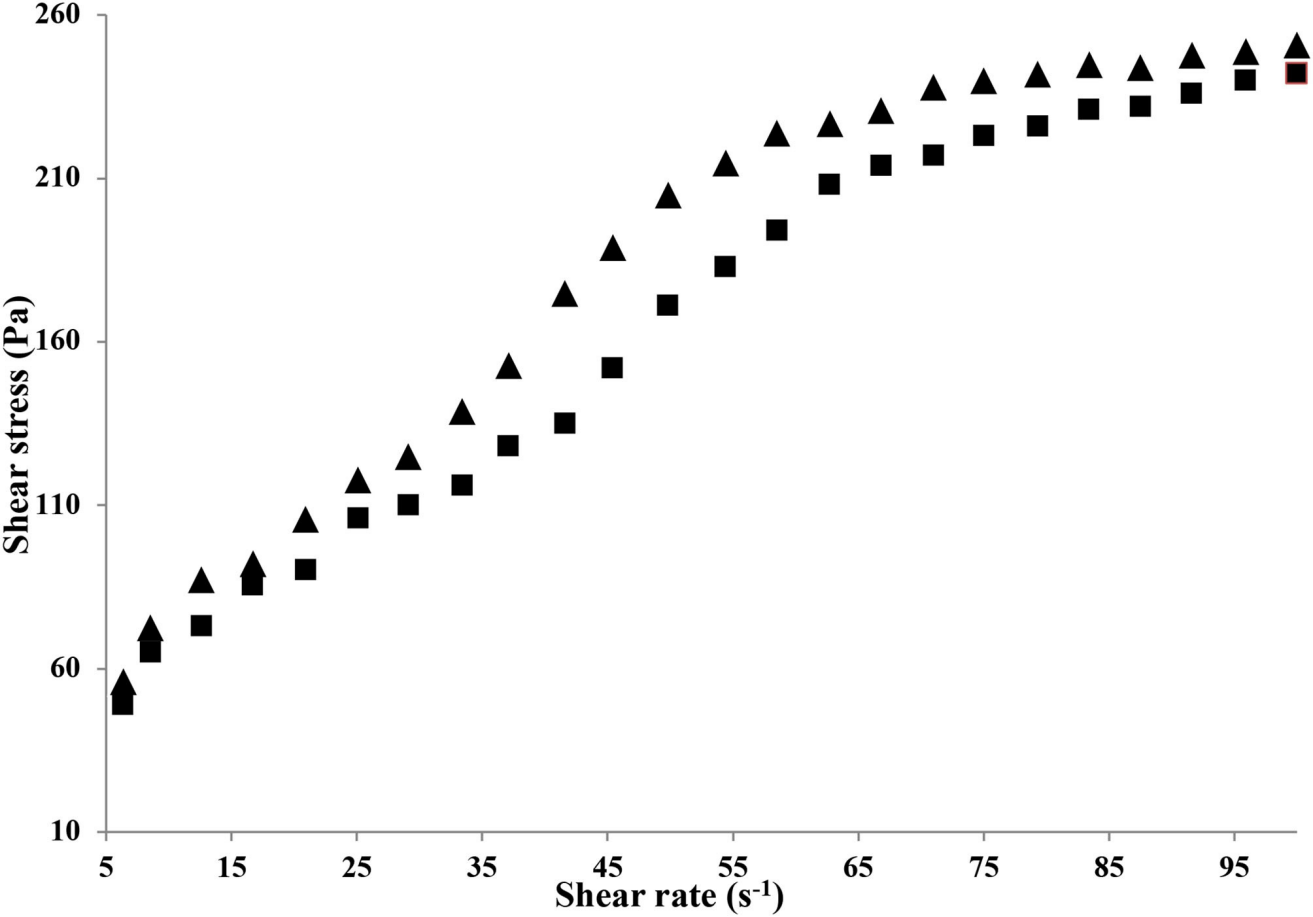
Flow behavior of HAD (▴) and FD (■) gelatin samples.

The flow behavior of HAD and FD gelatin solution is shown in Figure 3. The flow behavior of both HAD and FD gelatin solutions (6.67%) was best expressed by the Herschel–Bulkley model with the R^2^ values of 0.962 and 0.960, respectively. This model suggests that the material behaves as shear thinning (Pseudoplastic) material once considerable yield stress (σ_0_) initiated the flow. Both HAD and FD gelatin solution (6.67%) exhibited yield stress (56.1 and 49 pa) values, respectively, and thereafter showed pseudoplastic behavior. It has been recognized that shear thinning represents irreversible structure break-down and the diminish in viscosity is owing to molecular alignments taking place within such substances. Similar behavior was also observed by Binsi et al. (29) from big eye snapper fish skin gelatin.

The consistency coefficients (*k*) were 37.94 and 31.68 for HAD and FD gelatin solutions, respectively. The (*n*) values (flow behavior index) of the samples were 0.104 and 0.418 which confirms its shear-thinning behavior. Sarbon et al. (19) reported that this decrease in viscosity may be owing to the gradual breakage of microstructure in the gelatin samples with increased applied shear rate. It was found that both gelatin solutions exhibited pseudoplastic non-Newtonian behavior due to the value of *n* <1. The extent of n value was the one that differentiated their degree of pseudo-plasticity, and the higher the consistency factor (k), the better the gel consistency with a lower *n* value (30).

#### Frequency Sweep

**Figure 4:**
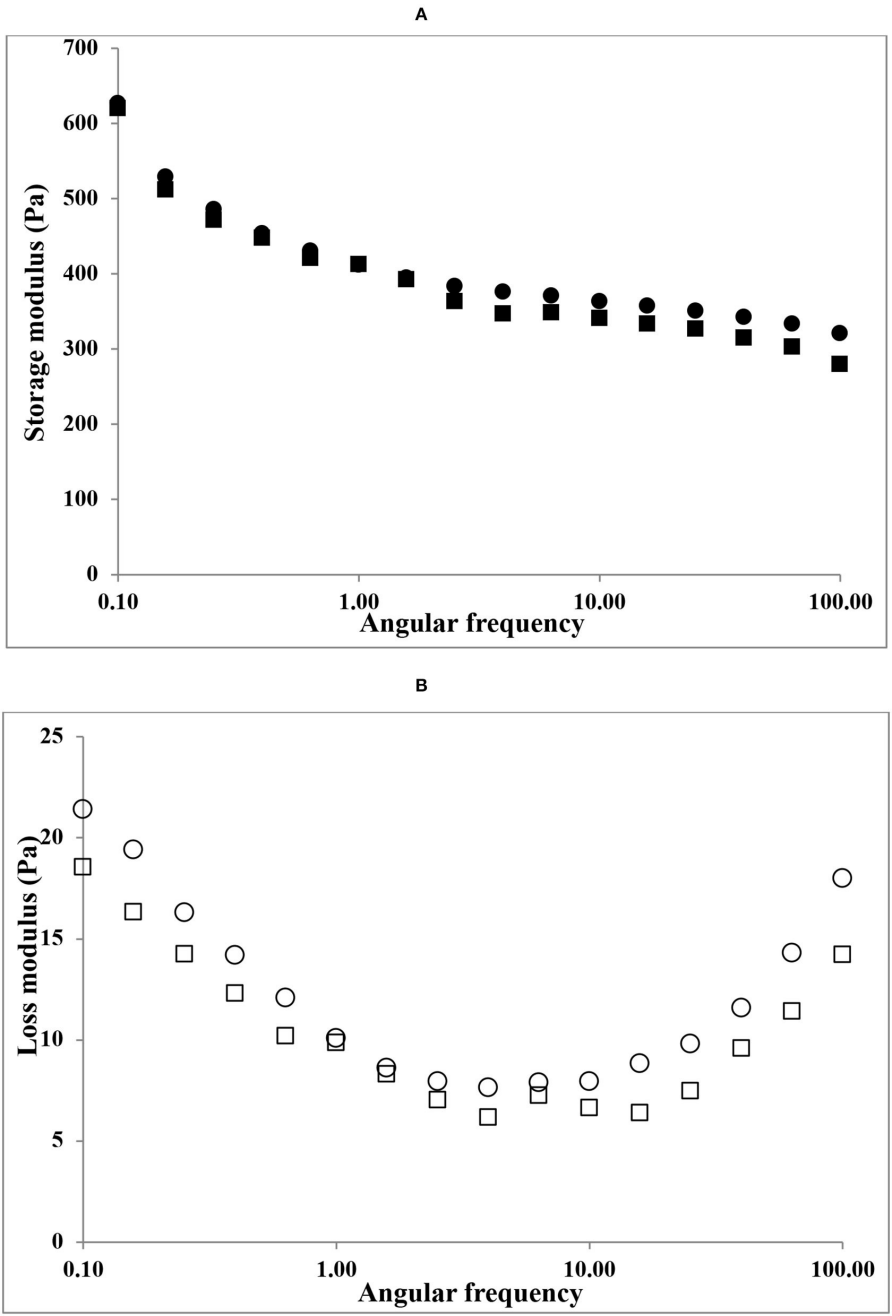
Frequency sweep plots of gelatin samples. **(A)** storage modulus (G') of HAD (■), FD (•), and **(B)** loss modulus (G”) of HAD (□) and FD (○) samples.

The viscoelasticity of gelatin gels (6.67%) by frequency sweep oscillatory sweep tests is shown in Figure 4. Both HAD and FD gelatin samples presented higher G′ than G″ during the 0.1–100 Hz frequency range. However, HAD gelatin solution shows higher storage modulus (G′) than FD gelatin samples. The higher storage modulus (G′) values over the whole frequency range of both the gelatin samples indicate an organized gel network with solid-like response to deformation (14). Eysturskarð* et al. (31) stated that there is a strong connection between the elastic modulus and the triple helices number; and the higher the G' values, the greater the number of triple-helical structures. They also suggested that the viscous modulus reveals the occurrence of dangling ends and loops connected to the networks or unrestricted chains, which contributes to energy dissipation *via* friction but not to the elastic complex strength.

Both HAD and FD gelatin samples showed similar loss modulus (G″) values. Similar findings in gelatin solution systems were also reported by Chandra and Shamasundar (32).

#### Temperature Sweep

**Figure 5:**
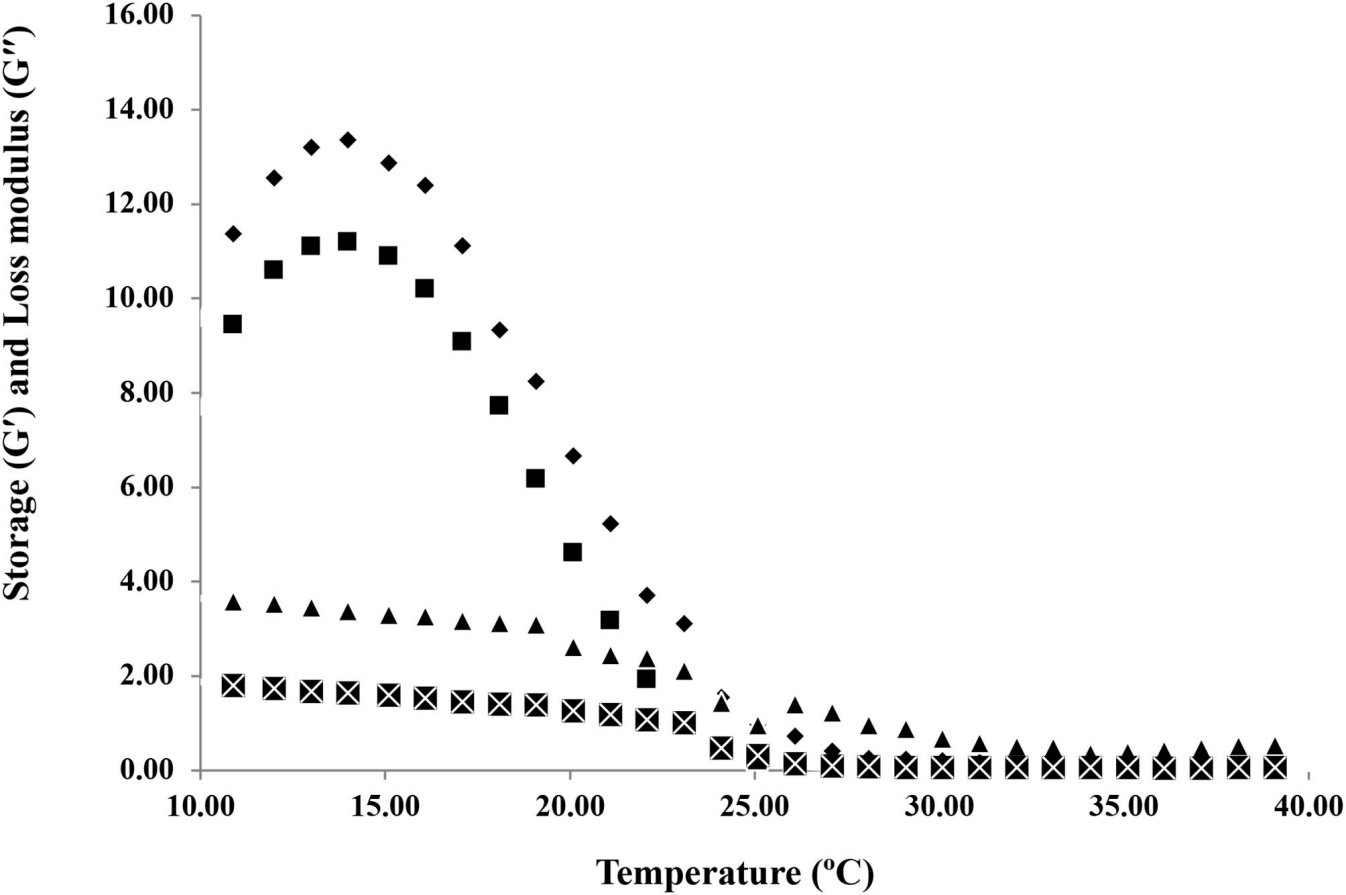
Temperature sweep tests of hot air dried and freeze-dried gelatin samples. Storage modulus (G') of HAD (♦), FD (■), and loss modulus (G”) of HAD (▴) and FD (**×**) samples.

The melting temperatures of HAD and FD gelatin samples were obtained by doing temperature sweep tests. The temperatures at which the elastic modulus (G′) and loss modulus (G′′) crossover through the heating process of gelatin samples from 10 to 40°C is shown in Figure 5. The viscoelastic properties of HAD and FD samples through heating at a constant heating rate (2°C/min) from 10 to 40°C were compared. The melting temperatures of HAD and FD gelatin samples were 25.10 and 23.10°C, respectively. The higher storage modulus value observed for HAD gelatin samples at low temperatures indicates its superior capacity to refold into a triple helical structure in junction zones (28). Generally, a higher value of G′ indicates superior thermostability, whereby a higher thermal change is required during heating and cooling. Overall, the melting temperature of HAD gelatin solution is higher than the FD gelatin solutions. This is also the reason for the greater gel strength of HAD gelatin samples than FD samples. Ninan et al. (33) stated that the higher setting and melting values of gelatin explain its applicability of gelatin. The increased bloom strength of gelatin gels is accompanied by the melting point escalation. Widyasari and Rawdkuen (17) reported that the chicken bones and cartilage gelatin have a melting point of 26.7°C, while the melting points of porcine and bovine gelatin range are between 20 and 25°C and 28 and 31°C, respectively. Kumar et al. (34) also reported that the melting temperature of croaker fish gelatin was 23.8°C.

### Texture Profile Analysis of Gelatin Gel

TPA is a two-bite test as the sample will be compressed twice by a suitable probe. The various TPA parameters of FD and HAD gelatin samples were shown in Table 2. It is evident from the results that HAD gelatin gel has higher hardness than the FD sample. Chandra and Shamasundar (11) reported hardness values of 14.60 N, 12.84 N, 8.41 N, and 7.05 N for gelatin gels of porcine, catla, mrigal, and rohu, respectively. The rate of material deformation under mechanical prosecution is related to internal structure strength and the internal bond breakdown difficulty is measured as cohesiveness (31). The FD gelatin gel has lower cohesiveness values than that of HAD. Adhesiveness is the force obligatory to overcome attraction between food and surface with which it comes in contact, such as teeth, tongue, and palate (32). The HAD gelatin gel has higher adhesiveness values than FD samples. The greater adhesiveness values indicate soft texture and can be used for the preparation of certain desserts (17). The springiness of substance is the rate at which the distorted sample returns to the original position after the elimination of deformation force (35). The HAD gelatin gel shows 0.0028 mm springiness value to FD samples having 0.0032 mm springiness value. The gumminess parameter is calculated as the hardness and cohesiveness of the product. The HAD gel shows higher gumminess values (16.86 N) than FD (3.72 N). Gumminess increases with an increase in product hardness (32). Chewiness is the chief texture characteristic of jelly products and is defined as the force obligatory to masticate the food for swallowing (33). The HAD gelatin gel shows higher chewiness values than FD samples. Similar to the gumminess parameter, chewiness increases as hardness increases (32).

### Functional Properties

#### Gel Strength (Bloom Value)

Gelatin is greatly accomplished in developing H-bonds with molecules of water as indicated in FTIR results, and hence forms stable three-dimensional gels, which is considered in terms of gel strength in food industries (13, 18). Typically, gelatin gels (6.67%) are classified on basis of gel strengths into various categories as higher bloom strength (200–300 g), medium bloom strength (100–200 g), and low bloom strength (50–100 g) gels (34). Different gel strengths of gelatins have different applicability in the food and pharmaceutical industry sectors. Gelatin is utilized in the production of soups, sauces, and meals for imparting smooth consistency (36). This is also utilized in low-fat spreads for acting as a binding agent. Gelatin hydrolysates have also been incorporated into various energy beverages for athletes (37). The bloom strength/gel strength values of HAD and FD gelatin samples were 276 and 251 g, respectively. Santana et al. (38) reported 119.1, 294.79, and 466.87 g bloom values of 4, 6.67, and 10% gelatin samples of chicken feet, respectively. Kanwate et al. (16) reported 67.54, 65.97, and 43.47 g bloom strength of freeze-dried, spray dried, and vaccum dried fish gelatin samples. The higher bloom/gel strength values of feet gelatin are attributed to its higher proline and hydroxyproline contents, which imparts stability to the gelatin structure through hydrogen bonding among H_2_O and OH hydroxyproline groups (39).

#### Foaming Capacity and Stability of Gelatin Samples

The gelatin shows desired to foam capacities by enhancing aqueous phase viscosity and thus decreasing surface tension at water-air interface (40). The FD gelatin sample foaming capacity was considerably higher (81.5%) than HAD (75.62%) gelatin samples. The lower foaming capacity for HAD sample proposed temperature-facilitated interactions between protein and water that inhibits the formation of foam. The various drying techniques may also result in diverse particle sizes of gelatin molecules that may also have an impact on foaming. The various authors reported that freeze-drying produces finer particles than vacuum drying. These results of the present study are in agreement with Kwak et al. (41), who reported a higher foaming capacity of FD gelatin than hot air or spray dried shark cartilage gelatin. Rasli and Sarbon (13) reported a foaming capacity of 176 and 80% of FD and vacuum dried chicken skin gelatin. Overall, proteins such as gelatin are promptly adsorbed by a recently created air-liquid interface through the bubbling and undergo unfolding and molecular reorganization at the interface. Therefore, it exhibited improved foaming capability than proteins that adsorb gradually and counter-attack unfolding (42). The FC of protein advances by the decrease of its surface tension, thereby exposing the gelatin to further hydrophobic residue (11).

**Figure 6:**
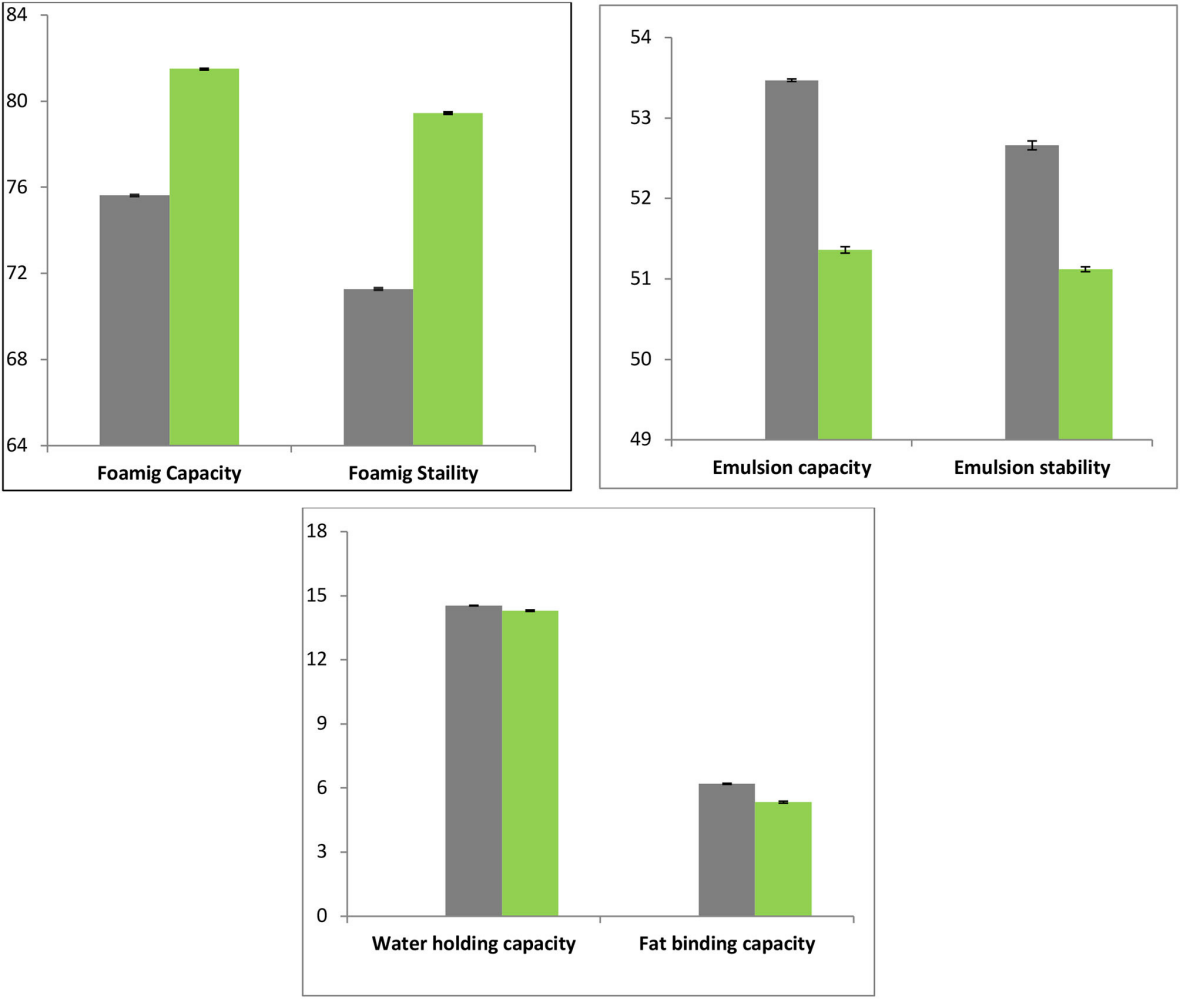
Physicochemical and functional properties of FD (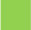) and HAD (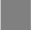) gelatin.

Figure 6 depicts the results for foaming stability of FD and HAD gelatin samples. The foaming stability of FD chicken feet gelatin (79.44%) was considerably higher than HAD (71.28%) samples. Sarbon et al. (19) stated that FD gelatin of poultry skin source had a greater quantity of hydrophobic groups like proline (13.42%) and alanine (10.08%), and hence greater foaming properties. The lower foaming stabilities of HAD gelatin can be owing to the lesser content of Aspartic and Glutamic acids (11). Aletor and Abiodun (43) also investigated the effects of dehydration on functional and protein solubility properties of vegetables and reported higher foaming stability and lower water absorption capacity of FD than sun-dried samples.

#### Water Holding Capacity

The FD and HAD gelatin samples do not differ considerably in WHC. The WHC of FD and HAD samples was 14.3 and 14.54 mL/g, respectively, as shown in Figure 6. Rasli and Sarbon (13) reported WHC of 15.6 mL/g for FD chicken skin gelatin and 15.37 mL/g for vacuum oven-dried samples. The WHC was supposed to be altered by the extent of hydrophilic amino acid residues (44). Thus, higher amounts of these amino acids contribute to higher water holding capacities. Sarbon et al. (19) stated that skin gelatin of poultry had higher amounts of hydrophilic amino acids like 5.84% glutamine, 5.57% arginine, 0.30% histidine, and 12.13% hydroxyproline contents. Rasli and Sarbon (13) also stated that the WHC of FD samples was greater (15.6 mL/g) than vacuum dried samples (15.37 mL/g) of gelatin, which was owing to dissimilarity in the hydrophilic contents.

#### Oil Binding Capacity

The OBC is an important property that is correlated to gelatin texture and depends on the interaction among oil and gelatin components (3). The fat binding capacity of FD chicken feet gelatin was 5.34 mL/g and that of HAD sample was 6.2 mL/g as shown in Figure 6. The HAD chicken feet gelatin has higher OBC which is likely owing to hydrophilic group exposer as compared to FD gelatin samples. The mechanism was supported by Chandra and Shamasundar and Ktari et al. (11, 44) who postulated that the hot-air drying process caused exposure of hydrophobic residues in the gelatin recovered from shark cartilage leading to its higher oil binding capacity. Jain and Anal (45) reported 2.5–4.4 mL/g FBC of eggshell protein and Dhakal et al. (46) reported 5.3 mL/g FBC of chicken feet collagen, which is in agreement with the results of the present study.

#### Emulsion Capacity and Emulsion Stability

Emulsification properties are used to examine the performance of proteinaceous substances in food emulsions. The EC of gelatin pronounces its capability to form adsorption films around oil/fat globules and to diminish the interfacial tension on the oil-water interface. The ES refers to the capability of an emulsion droplet to persist in a dispersed state and prevent separation by coalescing and creaming (47). The gelatin being amphoteric owing to hydrophobic/hydrophilic regions on the peptide chains permits it to assist as an emulsifier in numerous foods (47).

The emulsion capacity and stability of HAD and FD chicken feet gelatin are shown in Figure 6. The emulsion capacity of HAD sample was higher (53.47%) than the FD sample (51.36 %). Emulsion stability of HAD and FD samples was 52.66 and 51.12%, respectively, as presented in Figure 6. The hydrophobic regions of gelatin protein are the crucial factors to confine at the interface. Kanwate et al. (16) reported that drying techniques directly influenced the emulsification properties. Nagarajan et al. (48) also reported variation in emulsion stability and capacity of gelatin obtained from the splendid squid skin at diverse temperatures.

## Conclusion

Poultry byproducts (chicken feet) are excellent sources of gelatin protein, which can be utilized for innumerable purposes in the industrial sector. The present investigation was conducted to recover gelatin from chicken feet and evaluate the effect of the FD and HAD process on the gelatin's physicochemical and functional properties. There was no substantial variance in the yield of gelatin for HAD and FD methods. The FD samples showed higher lightness and lower redness values compared to HAD samples. Both the dried method samples showed a lower UV transmission rate. The FD and HAD samples showed peaks in amide regions namely, amide-A, amide-I, amide-II, and amide-III regions. Both HAD and FD gelatin solution showed yield stress and thereafter shear thinning behavior. Gelatin can be utilized as stabilizing, foaming, gelling, and texturizing agents in various food and non-food products. The HAD gelatin has higher gel strength than that of FD; however, reverse results were obtained when comparing the FC and FS of the gelatin samples. There was not a significant effect of drying techniques on the water holding capacity of gelatin; however, the emulsion capacity, stability, and oil binding capacity of HAD gelatin were higher than FD gelatin. Although both the dried gelatin powders have functionality in the food, pharmaceutical, and packaging industries, HAD gelatin can be the essential ingredient in food products where phase separation is the main problem. The FD gelatin can be utilized for foam food products for stabilizing the foam. Hence, these drying methods should be commercialized for the production of vulnerable gelatin for industrial applicability in numerous industrial sectors in the future.

## Data Availability Statement

The original contributions presented in the study are included in the article/Supplementary Material, further inquiries can be directed to the corresponding author/s.

## Author Contributions

JR: experiments, analysis, and initial draft. SDM: review and editing. AD: review. TA: analysis and review. HM: review, editing, verification of data, and design of experiments. FB: review and verification of data. BD: review, editing, and design of experiments. All authors contributed to the article and approved the submitted version.

## Funding

The authors would like to acknowledge the NPIU and TEQIP-III (MHRD Govt of India) for their faculty and financial support to the Food Technology Department, IUST Kashmir 192122 India.

## Conflict of Interest

The authors declare that the research was conducted in the absence of any commercial or financial relationships that could be construed as a potential conflict of interest.

## Publisher's Note

All claims expressed in this article are solely those of the authors and do not necessarily represent those of their affiliated organizations, or those of the publisher, the editors and the reviewers. Any product that may be evaluated in this article, or claim that may be made by its manufacturer, is not guaranteed or endorsed by the publisher.

## References

[1] Muduli S Champati A Popalghat HK Patel P Sneha KR. Poultry waste management: an approach for sustainable development. Int J Adv Sci Res. (2018) 4:8–14. Available online at: http://www.allscientificjournal.com/32069193

[2] Khan MR Sadiq MB Mehmood Z. Development of edible gelatin composite films enriched with polyphenol loaded nanoemulsions as chicken meat packaging material. CyTA J Food. (2020) 18:137–46. 10.1080/19476337.2020.1720826

[3] Xu M Wei L Xiao Y Bi H Yang H Du Y. Physicochemical and functional properties of gelatin extracted from Yak skin. Int J Biol Macromol. (2017) 95:1246–53. 10.1016/j.ijbiomac.2016.11.020 27838417

[4] Omar WHW Sarbon NM. Effect of drying method on functional properties and antioxidant activities of chicken skin gelatin hydrolysate. J Food Sci Technol. (2016) 53:3928–38. 10.1007/s13197-016-2379-5 28035148 PMC5156635

[5] Razali AN Amin AM Sarbon NM. Antioxidant activity and functional properties of fractionated cobia skin gelatin hydrolysate at different molecular weight. Int Food Res J. (2015) 22:651–60. Available online at: http://www.ifrj.upm.edu.my

[6] Nowak D Jakubczyk E. The freeze-drying of foods—The characteristic of the process course and the effect of its parameters on the physical properties of food materials. Foods. (2020) 9:1488. 10.3390/foods9101488 33080983 PMC7603155

[7] Chakka AK Muhammed A Sakhare PZ Bhaskar N. Poultry processing waste as an alternative source for mammalian gelatin: extraction and characterization of gelatin from chicken feet using food grade acids. Waste Biomass Valorization. (2017) 8:2583–93. 10.1007/s12649-016-9756-1

[8] Kanwate BW Kudre TG. Effect of various acids on physicochemical and functional characteristics of gelatin from swim bladder of rohu (Labeo rohita). J Food Sci Technol. (2017) 54:2540–50. 10.1007/s13197-017-2699-0 28740312 PMC5502050

[9] Hazirah MN Isa MIN Sarbon NM. Effect of xanthan gum on the physical and mechanical properties of gelatin-carboxymethyl cellulose film blends. Food Packaging Shelf Life. (2016) 9:55–63. 10.1016/j.fpsl.2016.05.008

[10] Rasid NAM Nazmi NNM Isa MIN Sarbon NM. Rheological, functional and antioxidant properties of films forming solution and active gelatin films incorporated with *Centella asiatica* (L.) urban extract. Food Packaging Shelf Life. (2018) 18:115–24. 10.1016/j.fpsl.2018.10.002

[11] Chandra M V Shamasundar BA. Texture profile analysis and functional properties of gelatin from the skin of three species of fresh water fish. Int J Food Properties. (2015) 18:572–84. 10.1080/10942912.2013.845787

[12] Sathe SK Deshpande SS Salunkhe DK. Functional properties of lupin seed (Lupinus mutabilis) proteins and protein concentrates. J Food Sci. (1982) 47:491–7. 10.1111/j.1365-2621.1982.tb10110.x

[13] Rasli HI Sarbon NM. Effects of different drying methods on the rheological, functional and structural properties of chicken skin gelatin compared to bovine gelatin. Int Food Res J. (2015) 22:584–92. Available online at: http://www.ifrj.upm.edu.my

[14] Shahidi F Han X-Q Synowiecki J. Production and characteristics of protein hydrolysates from capelin (Mallotus villosus). Food Chem. (1995) 53:285–93. 10.1016/0308-8146(95)93934-J

[15] Bichukale AD Koli JM Sonavane AE Vishwasrao V V Pujari KH Shingare PE. Functional properties of gelatin extracted from poultry skin and bone waste. Int J Pure Appl Biosci. (2018) 6:87–101. 10.18782/2320-7051.6768

[16] Kanwate BW Ballari R V Kudre TG. Influence of spray-drying, freeze-drying and vacuum-drying on physicochemical and functional properties of gelatin from Labeo rohita swim bladder. Int J Biol Macromol. (2019) 121:135–41. 10.1016/j.ijbiomac.2018.10.015 30290261

[17] Widyasari R Rawdkuen S. Extraction and characterization of gelatin from chicken feet by acid and ultrasound assisted extraction. Food Appl Biosci J. (2014) 2:85–97. Available online at: 10.14456/fabj.2014.7

[18] Almeida PF Lannes SCdS. Extraction and physicochemical characterization of gelatin from chicken by-product. J Food Process Eng. (2013) 36:824–33. 10.1111/jfpe.12051

[19] Sarbon NM Badii F Howell NK. Preparation and characterisation of chicken skin gelatin as an alternative to mammalian gelatin. Food Hydrocoll. (2013) 30:143–51. 10.1016/j.foodhyd.2012.05.009

[20] Shyni K Hema GS Ninan G Mathew S Joshy CG Lakshmanan PT. Isolation and characterization of gelatin from the skins of skipjack tuna (Katsuwonus pelamis), dog shark (Scoliodon sorrakowah), and rohu (Labeo rohita). Food Hydrocoll. (2014) 39:68–76. 10.1016/j.foodhyd.2013.12.008

[21] Singh S Singh K Singh B. Effect of drying techniques on physic-chemical & functional characteristics of gelatins from catla skins and its application. Pharma Inovat J. (2020) 9:11–7. Available online at: http://www.thepharmajournal.com/

[22] da Silva Araújo C Pino-Hernández E Batista S Thayse J Sarkis Peixoto Joele MR de Arimateia Rodrigues do Rego J. Optimization of fish gelatin drying processes and characterization of its properties. Sci Rep. (2021) 11:1–14. 10.1038/s41598-021-99085-3 34667229 PMC8526659

[23] Songchotikunpan P Tattiyakul J Supaphol P. Extraction and electrospinning of gelatin from fish skin. Int J Biol Macromol. (2008) 42:247–55. 10.1016/j.ijbiomac.2007.11.005 18207233

[24] Kim B-S Oh B-J Lee J-H Yoon YS Lee H-I. Effects of various drying methods on physicochemical characteristics and textural features of yellow croaker (Larimichthys Polyactis). Foods. (2020) 9:196. 10.3390/foods9020196 32075217 PMC7073827

[25] Rather JA Makroo HA Showkat QA Majid D Dar BN. Recovery of gelatin from poultry waste: characteristics of the gelatin and lotus starch-based coating material and its application in shelf-life enhancement of fresh cherry tomato. Food Packaging Shelf Life. (2022) 31:100775. 10.1016/j.fpsl.2021.100775

[26] Ahmad M Hani NM Nirmal NP Fazial FF Mohtar NF Romli SR. Optical and thermo-mechanical properties of composite films based on fish gelatin/rice flour fabricated by casting technique. Progress Organic Coat. (2015) 84:115–27. 10.1016/j.porgcoat.2015.02.016

[27] Hosseini SF Rezaei M Zandi M Ghavi FF. Preparation and functional properties of fish gelatin–chitosan blend edible films. Food Chem. (2013) 136:1490–5. 10.1016/j.foodchem.2012.09.081 23194553

[28] Al-Saidi G Rahman MS Al-Alawi A Guizani N. Thermal characteristics of gelatin extracted from shaari fish skin: effects of extraction conditions. J Therm Anal Calorim. (2011) 104:593–603. 10.1007/s10973-010-1240-8

[29] Binsi PK Shamasundar BA Dileep AO Badii F Howell NK. Rheological and functional properties of gelatin from the skin of Bigeye snapper (*Priacanthus hamrur*) fish: influence of gelatin on the gel-forming ability of fish mince. Food Hydrocoll. (2009) 23:132–45. 10.1016/j.foodhyd.2007.12.004

[30] Sinthusamran S Benjakul S Kishimura H. Characteristics and gel properties of gelatin from skin of seabass (Lates calcarifer) as influenced by extraction conditions. Food Chem. (2014) 152:276–84. 10.1016/j.foodchem.2013.11.109 24444937

[31] Eysturskarð J Haug IJ Elharfaoui N Djabourov M Draget KI. Structural and mechanical properties of fish gelatin as a function of extraction conditions. Food Hydrocoll. (2009) 23:1702–11. 10.1016/j.foodhyd.2009.01.008

[32] Chandra MV Shamasundar BA. Rheological properties of gelatin prepared from the swim bladders of freshwater fish Catla catla. Food Hydrocoll. (2015) 48:47–54. 10.1016/j.foodhyd.2015.01.022

[33] Ninan G Joseph J Abubacker Z. Physical, mechanical, and barrier properties of carp and mammalian skin gelatin films. J Food Sci. (2010) 75:E620–6. 10.1111/j.1750-3841.2010.01851.x 21535597

[34] Kumar DP Chandra M V Elavarasan K Shamasundar BA. Structural properties of gelatin extracted from croaker fish (Johnius sp) skin waste. Int J Food Properties. (2017) 20:S2612–25. 10.1080/10942912.2017.1381702

[35] Gómez-Guillén MC Giménez B López-Caballero ME al Montero MP. Functional and bioactive properties of collagen and gelatin from alternative sources: a review. Food Hydrocoll. (2011) 25:1813–27. 10.1016/j.foodhyd.2011.02.007

[36] Hamedi F Mohebbi M Shahidi F Azarpazhooh E. Ultrasound-assisted osmotic treatment of model food impregnated with pomegranate peel phenolic compounds: mass transfer, texture, and phenolic evaluations. Food Bioprocess Technol. (2018) 11:1061–74. 10.1007/s11947-018-2071-z

[37] Mutlu C Tontul SA Erbaş M. Production of a minimally processed jelly candy for children using honey instead of sugar. LWT. (2018) 93:499–505. 10.1016/j.lwt.2018.03.064

[38] Santana JCC Gardim RB Almeida PF Borini GB Quispe APB Llanos SA V. Valorization of chicken feet by-product of the poultry industry: high qualities of gelatin and biofilm from extraction of collagen. Polymers. (2020) 12:529. 10.3390/polym12030529 32121646 PMC7182801

[39] Calvarro J Perez-Palacios T Ruiz J. Modification of gelatin functionality for culinary applications by using transglutaminase. Int J Gastronomy Food Sci. (2016) 5:27–32. 10.1016/j.ijgfs.2016.11.001

[40] Saenmuang S Phothiset S Chumnanka C. Extraction and characterization of gelatin from black-bone chicken by-products. Food Sci Biotechnol. (2020) 29:469–78. 10.1007/s10068-019-00696-4 32296557 PMC7142178

[41] Kwak K Cho S Ji C Lee Y Kim S. Changes in functional properties of shark (Isurus oxyrinchus) cartilage gelatin produced by different drying methods. Int J Food Sci Technol. (2009) 44:1480–4. 10.1111/j.1365-2621.2007.01603.x

[42] Phillips GO Williams PA. Handbook of Food Proteins. Elsevier. (2011).

[43] Aletor O Abiodun AR. Assessing the effects of drying on the functional properties and protein solubility of some edible tropical leafy vegetables. Res J Chem Sci. (2013) 3:20–6. Available online at: http://www.iosrjournals.org/

[44] Ktari N Bkhairia I Jridi M Hamza I Riadh BS Nasri M. Digestive acid protease from zebra blenny (*Salaria basilisca*): characteristics and application in gelatin extraction. Food Res Int. (2014) 57:218–24. 10.1016/j.foodres.2014.01.041

[45] Jain S Anal AK. Optimization of extraction of functional protein hydrolysates from chicken egg shell membrane (ESM) by ultrasonic assisted extraction (UAE) and enzymatic hydrolysis. LWT Food Sci Technol. (2016) 69:295–302. 10.1016/j.lwt.2016.01.057

[46] Dhakal D Koomsap P Lamichhane A Sadiq MB Anal AK. Optimization of collagen extraction from chicken feet by papain hydrolysis and synthesis of chicken feet collagen based biopolymeric fibres. Food Biosci. (2018) 23:23–30. 10.1016/j.fbio.2018.03.003

[47] Damodaran S. Protein-stabilized foams and emulsions. In: Food Proteins and Their Applications. Boca Raton, FL: CRC Press. (2017). p. 57–110.

[48] Nagarajan M Benjakul S Prodpran T Songtipya P Kishimura H. Characteristics and functional properties of gelatin from splendid squid (Loligo formosana) skin as affected by extraction temperatures. Food Hydrocoll. (2012) 29:389–97. 10.1016/j.foodhyd.2012.04.001

