# Supplementary figures and images for: Extraction of Gelatin From Poultry Byproduct: Influence of Drying Method on Structural, Thermal, Functional, and Rheological Characteristics of the Dried Gelatin Powder
Source: Front Nutr. 2022 Jun 10;9:895197. doi: 10.3389/fnut.2022.895197 (PMC9226779; doi:10.3389/fnut.2022.895197)

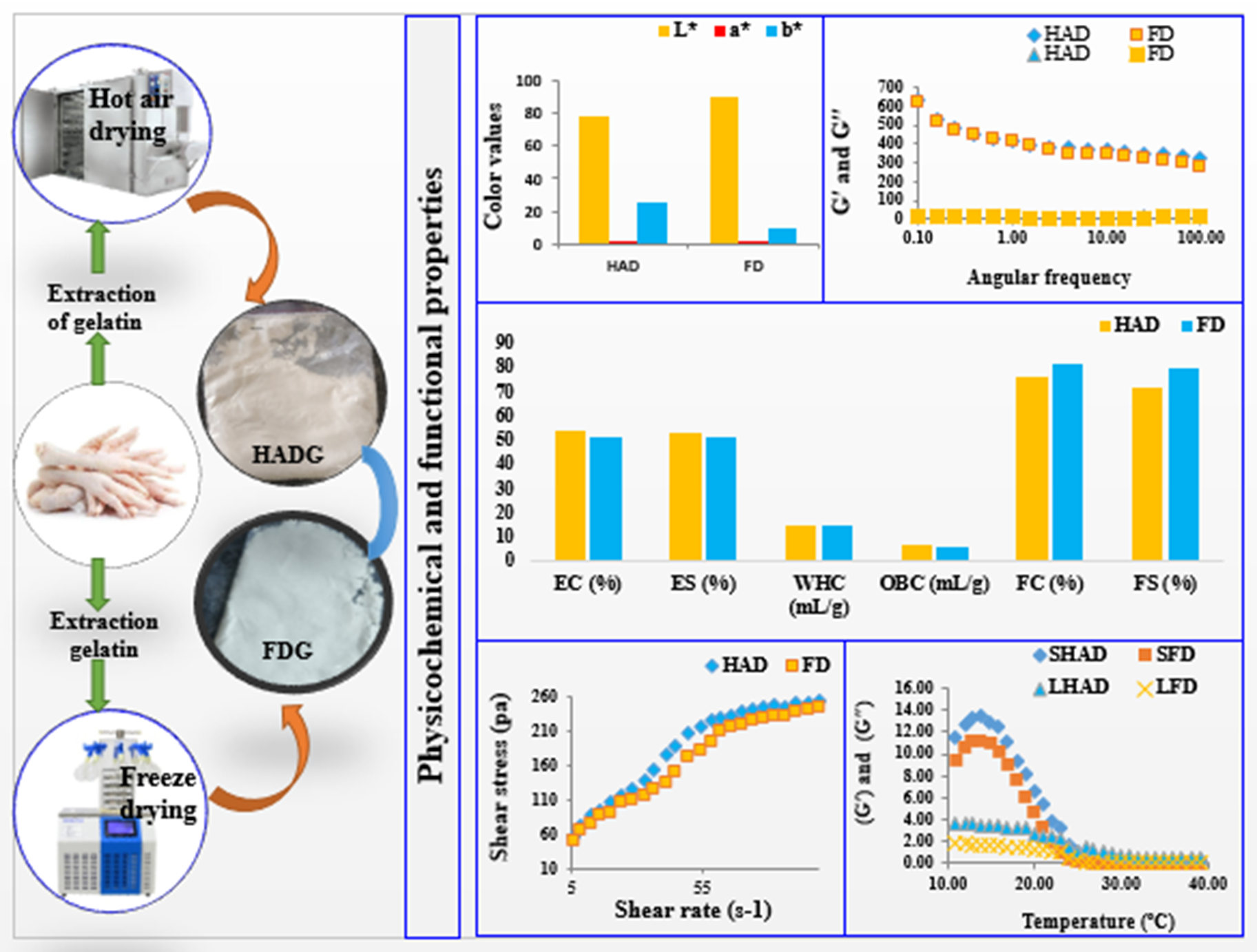

Supplement: Supplementary file 1 [file Image_1.PNG]
